# Dataset on the ketonisation of pure propionic acid and its mixture with pyrolysis bio-oil over metal oxide catalysts into 3-pentanone a biofuel precursor

**DOI:** 10.1016/j.dib.2025.111898

**Published:** 2025-07-18

**Authors:** Jude A. Onwudili, Abarasi Hart, Himanshu Patel, Eyup Yildirir

**Affiliations:** Energy and Bioproducts Research Institute, College of Engineering and Physical Sciences, Aston University, Aston Triangle, Birmingham B4 7ET, United Kingdom

**Keywords:** Pyrolysis, Bio-oil upgrading, Carboxylic acids, Propionic acid, Ketonisation, 3-pentanone, Biofuel

## Abstract

Biomass-derived compounds and pyrolysis bio-oils would play a crucial role in meeting the globally goal towards decarbonization of the aviation industry through sustainable aviation fuel (SAF). The carbon number of carboxylic acids abundant in biomass pyrolysis bio-oils is mostly within C_1_-C_3_, which falls short of gasoline and aviation fuels hydrocarbon range. These carboxylic acids require C-C coupling via ketonisation and then, aldol condensation to produce elongated and branched chain precursors with similar carbon-chain to match gasoline and jet fuel (C_6_-C_16_). This dataset was obtained from solvent-free ketonisation of propionic acid, one of the abundant short-chain carboxylic acids found in biomass pyrolysis bio-oils using synthesised ZrO_2_, SiO_2_-ZrO_2_, and SiO_2_ catalysts at 300-400 °C for 0-210 min in a stirred batch reactor. The data elucidates the different side reactions such as isomerisation, alkylation, cleavage of C-C bond, and cross ketonisation resulting in isomeric, straight, and branched ketones (C_4_-C_7_) with selectivity of about 9.2%, limiting selectivity towards 3-pentanone, the propionic acid self-ketonisation product. The influence of these side reactions during the ketonisation process was shown by the data on conversion of propionic acid, selectivities, and yields of 3-pentanone and other ketones, allowing performance evaluation of the oxide catalysts. The data indicates that these side reactions are dependent on reaction temperature, reaction time, and amphoteric nature of the catalyst. The data provides support for the robustness, activeness, and selectiveness of ZrO_2_ in the ketonisation of short-chain carboxylic acids into fuel-range ketone precursors in the presence of 50 wt% bio-oil. The industrial concept of bio-oil upgrading via ketonisation is reinforced by the data on propionic acid plus bio-oil reactions and hydrodeoxygenation.

## Graphical abstract

**Figure:**
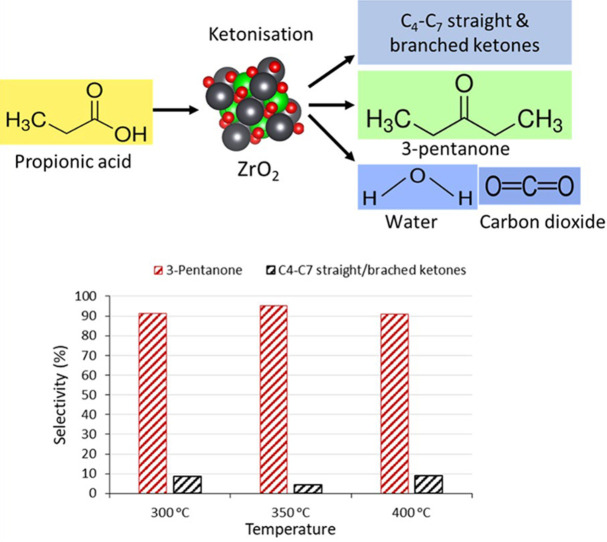

Specifications Table

**Table:** 

Subject	Biofuel
Specific subject area	Chemical reaction via ketonisation of carboxylic acids into elongated ketones biofuel precursor
Data format	Raw, analysed
Type of data	Tables, MS Excel Spreadsheet
Data collection	Textural properties data on the synthesised ZrO_2_, SiO_2_, and SiO_2_–ZrO_2_ oxides were obtained using nitrogen sorption technique (Quantachrome Instruments NOVA 4200, Quantachrome UK Limited), and their crystallinity and phase composition by using X-ray diffraction (XRD) technique (Bruker D8 Advance A25, Bruker AXS GmbH, Karlsruhe, Germany). Data on selectivity of ketonisation products were obtained from the compositional analysis of the liquid products using gas chromatograph-mass spectrometer, GC-MS (Shimadzu GCMS – QP2010 SE). Data on propionic acid conversion and yields of 3-pentanone quantification and calibration of GC-MS liquid analysis.
Data source location	Energy and Bioproducts Research Institute, College of Engineering and Physical Sciences, Aston University, Aston Triangle, Birmingham B4 7ET, United Kingdom
Data accessibility	Repository name: Mendeley Data Data identification number: 10.17632/bf6vpy66bw.1 Direct URL to data: https://data.mendeley.com/datasets/bf6vpy66bw/1 Instructions for accessing these data: The above URL can be used to access the data.
Related research article	[1]

## 1 Value of the Data

• This dataset contains the range of ketones produced from the ketonisation of propionic acid ketonisation over oxide catalysts, which are not usually reported in carboxylic acid ketonisation.
• This dataset provides insight into the extent of side reactions occurring during the ketonisation of propionic acid over metal oxide catalysts.
• This dataset can also help determine the effect of process variables such as reaction temperature and time on the extent of side reactions which lowers selectivity towards the desired 3-pentanone product.
• The comprehensive spectra of compounds identified by GC-MS and their calculated selectivity reveal to researchers the different type of reactions occurring during propionic acid ketonisation over metal oxide catalysts.
• This dataset supports efforts in catalyst development for bio-oil upgrading via ketonisation route, and technoeconomic studies toward industrial scale process.
• The experimental dataset provided in this study will benefit research scientists and sustainable fuel industry stakeholders on the valuable insights and knowledge on novel catalytic upgrading of pyrolysis bio-oil and its model compounds. The dataset could enable the development of cheaper, active and selective catalysts for minimising side reactions in the multicomponent reactions involved during bio-oil upgrading. Ultimately, the results could lead to improved conversion efficiencies while enhancing the economic viability, scalability and environmental sustainability of producing replacement hydrocarbon-rich liquid fuels from lignocellulosic biomass.

## 2 Background

This dataset was collected for the purpose of gaining insight into upgrading significant portions of short-chain carboxylic acids found in bio-oils derived from the pyrolysis of lignocellulosic biomass into liquid fuel range precursors via ketonisation C-C coupling [1,2]. A model short-chain carboxylic acid, propionic acid was ketonised into 3-pentanone over synthesised oxides ZrO_2_, SiO_2_, and SiO_2_–ZrO_2_ catalysts using a stirred 100 mL batch reactor (Parr Instrument Company, IL, USA) between 300 °C and 400 °C reaction temperature, 0 to 210 min reaction time, and 15/1 feedstock-to-catalyst ratio at 10 bar initial pressure of nitrogen/hydrogen. The quantification of the liquid product was carried out to determine the conversion of propionic acid, the yield and selectivity of 3-pentanone using developed calibration curves and GC-MS compositional areas.

Although ketonisation of carboxylic acids found in pyrolysis bio-oil offers a pathway to elongating carbon chain length and deoxygenation of the product, it suffers from rapid deactivation of catalysts, due to structural changes because of reaction medium and conditions, in-situ produced water, carbon dioxide adsorption, or coke formation [1,3,4]. Likewise, the ketonisation of pure short-chain propionic acid into 3-pentanone is commonly faced with the challenges of catalyst deactivation and low selectivity towards desired ketone (e.g., 3-pentanone) as a result of competitive side reactions. Additionally, ketonisation of the carboxylic acids in the complex mixture of bio-oil, which is a potential source of propionic acid, is challenging due to its heterogeneity. Therefore, the stability, activity, and selectivity towards desired ketone product can be improved through catalyst design and optimise acid-base properties [1].

Using a propionic acid-to-bio-oil ratio of 50:50 (g/g), data on the effect of bio-oil compounds on the ketonisation of propionic acid over ZrO_2_ catalyst was obtained at the optimum reaction temperature of 350 °C and time 180 min in hydrogen atmosphere. The significance of this data lies in the demonstration of side reactions occurring during self ketonisation of propionic acid, and provides evidence to support that ZrO_2_ is robust, active, and selective toward ketonisation of propionic acid in the presence of other classes of organic compounds in bio-oils. This dataset, therefore, complement the article published in the literature [1], which focused mostly on 3-pentanone production. Furthermore, the data provides evidence that ketonisation of reactive short-chain carboxylic acids into C-C elongated ketones with partial deoxygenation is a plausible strategy for bio-oil upgrading [5,6]. A broad range of biofuel ketonic precursors are produced as evident in this data, elucidating their potential as building blocks for further chain elongation via aldol condensation into aviation fuel range precursors, and subsequent hydrodeoxygenation into jet biofuel.

## 3 Data Description

The dataset on the crystallinity, specific surface area and pore size distribution data of synthesised oxides ZrO_2_, SiO_2_, and SiO_2_–ZrO_2_ materials can be found in the “Sheet2” and “Sheet3". The dataset on Sheet2 represents the diffractogram of the XRD patterns of the synthesised single and mixed oxide catalysts showing the diffraction angle (2θ) and intensity of diffraction peaks. The dataset provides information about the crystallinity of synthesised catalyst structure, crystalline phases present, crystallite sizes, and crystal orientations. Whereas the nitrogen adsorption-desorption isotherms dataset presented in Sheet3, show the specific volume of adsorbed nitrogen gas molecules versus relative pressure, and volume of adsorbed against pore diameter. This dataset information is used to determine the synthesised catalysts' specific surface areas, pore structure, and pore size distribution.

The dataset also comprises data on the effect of reaction temperature and time on the selectively towards 3-pentanone and other ketones formed from side reactions. Sheet4 shows the peak areas of ketonisation products of propionic acid over ZrO_2_ catalyst at 350 °C reaction temperature, 10 bar initial hydrogen pressure, 15 g propionic acid, 1.0 g catalyst loading, and 500 rpm stirring rate for the different reaction time are tabulated. Peak areas of propionic acid ketonisation products over ZrO_2_ catalyst as a function of reaction temperature at 300 °C, 350 °C, and 400 °C when the reactor reaches the target temperature, denoted as reaction time "0" are presented in Sheet5. The different ketones produced from the ketonisation of propionic acid based on their peak areas shown in the GC-MS compositional dataset includes unconverted propionic acid, desired 3-pentanone and other ketones. In Sheet6, the dataset on the yields of liquid, gaseous, and solid (char) products as well as the conversion of propionic acid and the yields of 3-pentanone (desired liquid product) for ketonisation over the different catalysts ZrO_2_, SiO_2_, and SiO_2_–ZrO_2_ at 300 °C, 350 °C, and 400 °C reaction temperature and 180 min reaction time using 10 bar initial hydrogen pressure, 15 g propionic acid, 1.0 g catalyst loading, and 500 rpm stirring rate are presented. The dataset has been supplied separately in a Microsoft Excel spreadsheet. Data used for propionic acid conversions, yields and selectivity of 3-pentanone as a function of catalyst type is also included in the dataset “Sheet4-Sheet6”. The dataset in Sheet7 shows the peak areas of GC-MS of raw bio-oil, upgraded bio-oil, and reacted propionic acid+bio-oil (50/50 g/g) over ZrO_2_ catalyst at 350 °C reaction temperature, 10 bar initial hydrogen pressure, and 180 min reaction time. In Sheet7, the compounds present in the liquids (bio-oil, upgraded bio-oil, and reacted propionic acid+bio-oil) have been classified into C_1_-C_3_ carboxylic acids, >C_3_ carboxylic acids, aldehydes, alcohols, C_4_-C_7_ straight and branched ketones, >C_7_ and cyclic ketones, esters, ethers, and hydrocarbons. The GC-MS compositional analysis dataset on raw bio-oil, and the liquid products from upgraded bio-oil and ketonised bio-oil + propionic acid can be found in “Sheet7” in the dataset. Table 1 summarises the contents of the dataset and where they can be found.

**Table 1:** Contents of the provided dataset.

Sheet number	Sheet	Content
1	Content	Table of content
2	Oxide catalysts crystallinity and phase composition analysis	X-ray diffraction (XRD) data on the synthesised single and mixed oxide catalysts
3	Surface area and pore size analysis	Specific surface area and pore size distribution data of the synthesised single and mixed oxide catalysts
4	GC-MS composition analysis	Data for the selectivity of 3-pentanone and other ketones in the liquid product from propionic acid ketonisation as a function of time
5	GC-MS composition analysis	Data on the liquid product of propionic acid ketonisation, selectivity for 3-pentanone and other ketones as a function of reaction temperature
6	GC-MS quantification analysis	Conversions of propionic acid, yields and selectivity to 3-pentanone in the liquid product as a function of reaction temperature, catalyst oxide acidity/basicity, and reaction atmosphere
7	GC-MS composition analysis	Data on raw bio-oil, ketonised bio-oil+propionic acid (50/50), and upgraded bio-oil

**Fig 1:**
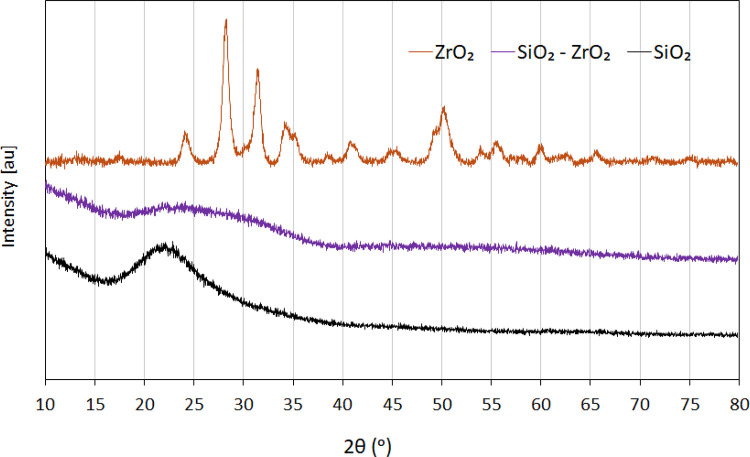
The XRD patterns of the synthesised single metal oxides (weak acid SiO_2_ and highly amphoteric ZrO_2_), and moderately amphoteric mixed metal oxides of SiO_2_-ZrO_2_ catalysts.

**Fig 2:**
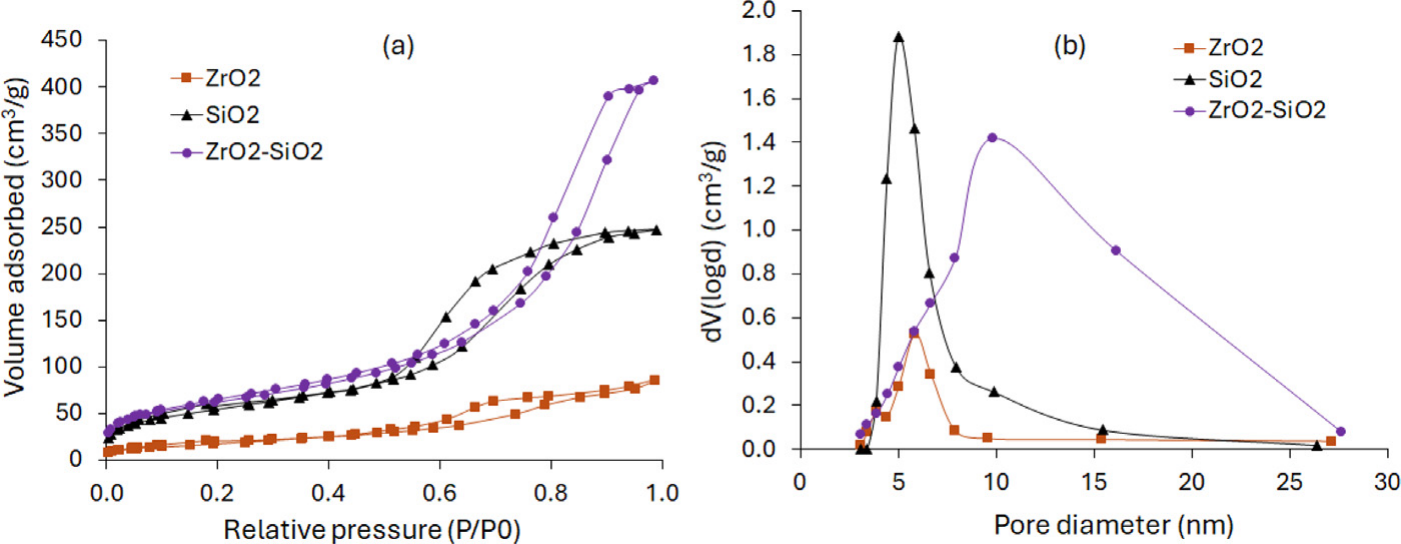
The catalysts textural properties: (a) nitrogen adsorption-desorption isotherms, and (b) pore size distribution.

The XRD and nitrogen sorption data profile are shown in Fig 1, Fig 2, which are designated in the dataset as “Sheet2 and Sheet3”, respectively. The selectivity data for 3-pentanone and other ketones from the ketonisation of propionic acid over ZrO_2_ catalyst is summarised in Table 2. This is provided in the sheet labelled “Sheet4” in the dataset. The effect of reaction temperature on the selectivity of 3-pentanone and other ketones are provided in the sheet labelled “Sheet5” in the dataset and summarised in Table 3. Here ‘0 min’ implies that the reactor heating jacket was removed and cooled once the reaction temperature has been attained.

**Table 2:** The selectivity of 3-pentanone and other ketones as a function of reaction time for ketonisation of propionic acid over ZrO_2_ at 350 °C temperature, under 10 bar initial nitrogen atmosphere.

Products	Selectivity (%)			
	0 (min)	60 (min)	120 (min)	180 (min)
2-Butanone	1.51	2.45	2.73	3.24
2-Pentene, 3-methyl-	0.00	0.17	0.34	0.09
2-Pentanone	0.00	0.09	0.20	0.09
3-Pentanone	95.10	93.39	90.53	92.85
3-Methyl-3-hexene	0.49	1.16	2.02	0.82
Propanoic acid, ethyl ester	0.29			
3-Pentanone, 2-methyl-	0.31	1.36	1.60	2.33
3-Hexanone			0.19	
3-Hexanone, 4-methyl-	0.42	0.55	1.09	0.58
3-Heptanone	0.22			
4-Hexen-3-one, 4-methyl-	0.26			
3-Heptanone, 4-methyl-			0.67	
5-Hepten-3-one, 5-methyl-	0.15			
1-Ethylhexyl propionate	0.24			
3-Heptanone, 5-ethyl-4-methyl-		0.27		
4-Hexen-3-one, 4,5-dimethyl-	0.76	0.16	0.22	
Carvenone	0.25	0.28	0.18

**Table 3:** The selectivity of 3-pentanone and other ketones as a function of reaction temperature in the ketonisation of propionic acid over ZrO_2_ at “0 min”, under 10 bar initial nitrogen atmosphere.

Products	Selectivity (%)		
	300 °C	350 °C	400 °C
2-Butanone	4.27	1.51	4.44
2-Pentene, 3-methyl-	0.35		0.36
2-Butanone, 3-methyl-	0.20		0.21
2-Pentanone	0.21		0.23
3-Pentanone	91.37	95.10	90.85
3-Methyl-3-hexene	0.82	0.49	0.79
Propanoic acid, ethyl ester		0.29	
3-Pentanone, 2-methyl-	1.61	0.31	1.69
3-Hexanone	0.23		0.25
3-Hexanone, 4-methyl-			0.56
3-Heptanone		0.22	
4-Hexen-3-one, 4-methyl-		0.26	
3-Heptanone, 4-methyl-	0.57		0.22
5-Hepten-3-one, 5-methyl-		0.15	
1-Ethylhexyl propionate		0.24	
3-Heptanone, 5-ethyl-4-methyl-			
4-Hexen-3-one, 4,5-dimethyl-	0.23	0.76	0.24
Carvenone	0.13	0.25	0.16

**Fig 3:**
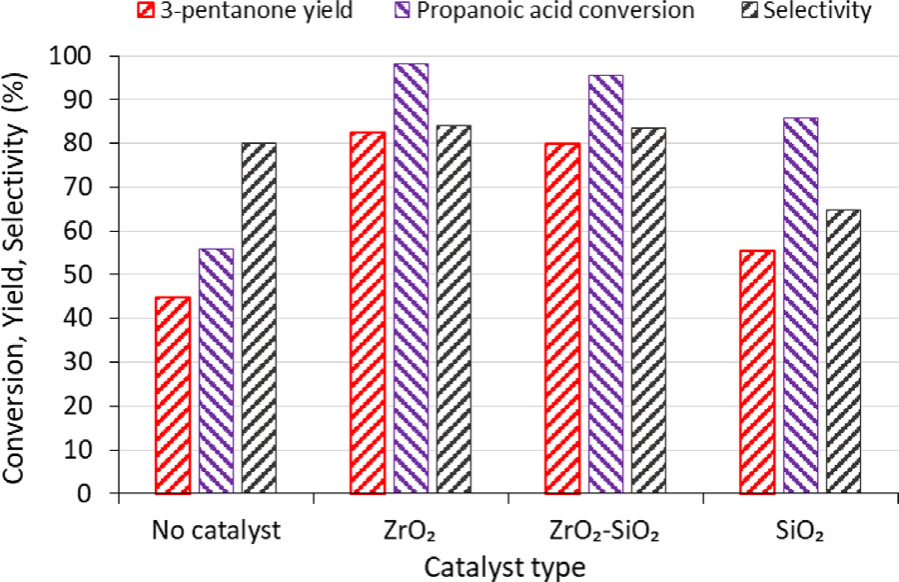
Conversion of propionic acid, yield and selectivity of 3-pentanone for both non-catalytic and SiO_2_, ZrO_2_-SiO_2_, and ZrO_2_ catalysed ketonisation at 350 °C reaction temperature, 180 min reaction time, and 15 g propionic acid starting with 10 bar initial hydrogen pressure.

**Fig 4:**
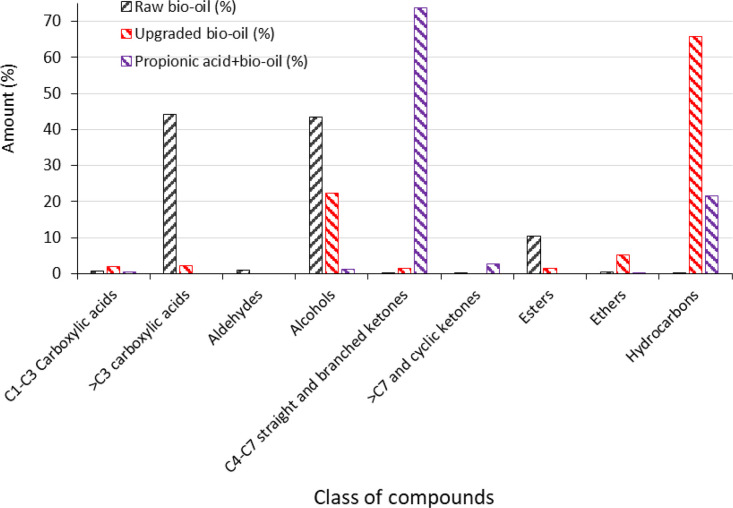
Effect of bio-oil on propionic acid conversion via ketonisation and products distribution (7.5 g bio-oil, 7.5 g propionic acid, 1 g ZrO_2_, 10 initial H_2_ pressure, 350°C temperature for 180 min).

Fig. 3 summarizes the data provided in “Sheet6” of the dataset for propionic acid conversions, yields and selectivity towards 3-pentanone for the different oxide catalysts synthesised. Products distribution data provided the GC-MS compositional analysis presented in “Sheet7” of the dataset for raw bio-oil, upgraded bio-oil, and ketonisation of 50/50 mixture of propionic acid and bio-oil are shown in Fig. 4.

## 4 Experimental Design, Materials and Methods

The experimental design is based on one factor at a time (OFAT) methodology. Propionic acid ketonisation was carried out using 100 mL stirred batch reactor (Parr Instrument Company, IL, USA), 15 g propionic acid, 1.0 g catalyst loading (ZrO_2_, SiO_2_, and SiO_2_–ZrO_2_), 500 rpm stirring rate, and pressurized initially to 10 bar nitrogen or hydrogen. The reaction time was optimized by experimenting at 350 °C from 0 to 210 min for step size of 60 min, and the optimization of reaction temperature at 180 min from 300 to 400 °C for 50 °C interval under nitrogen atmosphere. The effect of bio-oil was conducted using 50/50 weight mixture with propionic acid. The produced liquid after the ketonisation reaction was analysed using gas chromatograph-mass spectrometer (GC-MS) instruments (Shimadzu GCMS – QP2010 SE). Calibration curves prepared using 20, 40, 60, 80, and 100 µL of propionic acid/3-pentanone per 1.6 mL acetone (vol/vol) ratios was utilised to quantify the unconverted propionic acid and produced 3-pentanone [1]. The data were tabulated and analysed using Microsoft spreadsheets. The conversion and yield were calculated using (1), (2), while the selectivity of each product identified by the GC-MS liquid composition analysis was determine using Eq. 3.

(1) $$Conversion\;\left[\%\right]=\;\frac{\text{moles}\;\text{of}\;\text{propionic}\;\text{acid}\;\text{converted}}{\text{mole}\;\text{of}\;\text{propionic}\;\text{acid}\;\text{fed}\;\text{into}\;\text{the}\;\text{reactor}}\;\times \;100$$

(2) $$Product\;yield\;\left[\%\right]=\;\frac{Moles_{3-pentanone}}{Moles_{Propionic\;acid}}\;\times \;100$$

(3) $$Selectivity\;\left[\%\right]=\;\frac{\text{Peak}\;\text{Area}\;\text{of}\;\text{component}\;i}{\text{Total}\;\text{Peak}\;\text{Area}\;\text{of}\;\text{all}\;\text{Components}\;\text{in}\;\text{liquid}\;\text{product}}\;\times \;100$$

The oxide catalysts ZrO_2_ and SiO_2_ were synthesized via precipitation method, while co-precipitation was used for the mixed oxides (ZrO_2_-SiO_2_) at a ratio of 1:1, using sodium meta-silicate nanohydrate (Na_2_SiO_3_.9H_2_O), zirconyl chloride octahydrate (ZrOCl_2_·8H_2_O), aqueous solution of ammonium hydroxide, NH_4_OH (50 % vol/vol), and hydrochloric acid (HCl) [1]. The synthesised materials were oven-dried at 105 ^ο^C for 12 h and calcined at 500 °C for 4 h in air. The specific surface areas, pore size distributions and crystallinity were determined using Quantachrome Instruments NOVA 4200 and X-ray diffraction (XRD) technique (Bruker D8 Advance A25) [7].

## Limitations

The dataset is limited to self-ketonisation of propionic acid, whereas in a real and complex bio-oil mixture cross-ketonisation is expected to occur. Additionally, the size of dataset is not large enough statistical analysis. Further data beyond what has been presented is expected on the effect of bio-oil organic components on propionic acid ketonisation. This is required to further probe catalyst stability and performance which closely mimic real bio-oil upgrading. Kinetic modelling data on propionic acid ketonisation is required to fully grasp the reaction mechanism. The GC-MS peak areas could be used as a good indication of the concentrations of compounds contained in the liquid product from the ketonisation reaction. However, this method of calculating selectivity based on the peak areas of identified compounds by the GC-MS might not account for unidentified compounds.

## Ethics Statement

This work was strictly on chemical reaction experiments, and so does not involve human subjects, animal experiments, or any data collected from social media platforms.

## Funding

This work was supported by Innovate UK Energy Catalyst Round 8: Clean Energy - Experimental Development (Project Number 75521) and Innovate UK Energy Catalyst Round 9 – Mid Stage (Project Number 10047783).

## CRediT Author Statement

**Jude A. Onwudili:** Conceptualization, Project administration, Resources, Supervision, Validation, Visualization, Writing – original draft, Writing – review & editing; **Abarasi Hart**: Methodology, Investigation, Formal analysis, Data curation, Validation, Writing – original draft, Writing – review & editing; **Himanshu Patel:** Methodology, Investigation, Data curation, Formal analysis, Writing – review & editing. **Eyup Yildirir**: Methodology, Investigation, Data curation, Formal analysis, Writing – review & editing.

## Data Availability

Mendeley DataDataset on products selectivity on the effect of bio-oil on the ketonisation of propionic acid over metal oxide catalysts into 3-pentanone a biofuel precursor.xlsx (Original data).
